# Impaired noise adaptation contributes to speech intelligibility problems in people with hearing loss

**DOI:** 10.1038/s41598-024-80131-9

**Published:** 2024-11-20

**Authors:** Miriam I. Marrufo-Pérez, Milagros J. Fumero, Almudena Eustaquio-Martín, Enrique A. Lopez-Poveda

**Affiliations:** 1 Instituto de Neurociencias de Castilla y León, Universidad de Salamanca, Calle Pintor Fernando Gallego 1, 37007 Salamanca, Spain; 2 Instituto de Investigación Biomédica, Universidad de Salamanca, Salamanca, Spain; 3 Departamento de Cirugía, Facultad de Medicina, Universidad de Salamanca, Salamanca, Spain

**Keywords:** Hearing aids, Adaptation, Speech spectrum, Envelope, Temporal fine structure, Auditory system, Human behaviour

## Abstract

Understanding speech in noisy settings is harder for hearing-impaired (HI) people than for normal-hearing (NH) people, even when speech is audible. This is often attributed to hearing loss altering the neural encoding of temporal and/or spectral speech cues. Here, we investigated whether this difference may also be due to an impaired ability to adapt to background noise. For 25 adult hearing-aid users with sensorineural hearing loss, speech reception thresholds (SRTs) were measured for natural and tone-vocoded words embedded in speech-shaped noise (SSN). The stimuli were preceded or not by a 1-second adapting SSN precursor. Adaptation was calculated as the difference in SRT between the two precursor conditions. Corresponding data for 28 NH listeners were taken from a previously published study. SRTs were worse for HI listeners, confirming that hearing loss diminishes access to speech acoustic cues. Furthermore, noise adaptation was negatively correlated with the age-controlled hearing loss both for natural (rho=-0.56, *N* = 52, *p* < 0.001) and vocoded (rho=-0.45, *N* = 39, *p* = 0.002) words. Impaired adaptation contributed up to 10% of the SRT loss in HI listeners. We conclude that HI listeners suffer from poorer speech in noise recognition not only because of impaired access to speech acoustic cues but also because they are less able to adapt to background noise.

**Supplementary Information:**

The online version contains supplementary material available at 10.1038/s41598-024-80131-9.

## Introduction

Hearing loss affects millions of people worldwide and has a negative impact on many areas, including speech intelligibility. Hearing-impaired (HI) listeners show poorer speech recognition in noisy environments than do normal-hearing (NH) listeners even when speech is presented at suprathreshold levels^1–8^. This has prompted many studies to investigate the cause of non-audibility related deficits^9–11^ and led to the search for new sound-processing algorithms to improve speech-in-noise intelligibility^12,13^. To date, however, studies have overlooked that the greater difficulties experienced by HI listeners understanding speech in noisy environments could be partly due to an impaired ability to adapt to background noise. The aim of the present study was to investigate the potential negative impact of hearing loss on the ability of individuals to adapt to noise during speech recognition.

The ability of NH listeners to recognize words or syllables in noisy settings can improve as speech tokens are delayed from the onset of the noise. This has been interpreted as evidence that NH listeners adapt to the background noise^14–17^. Adaptation to noise occurs in pure tone detection^18^ (also known as “overshoot”), amplitude modulation (AM) detection^19,20^, syllable recognition^21^, and word recognition^16,17^. This adaptation has a time course of approximately 300–350 ms^18,22,23^ and its benefits can be quite large for NH listeners. For example, for speech presented at a fixed signal-to-noise ratio (SNR), adaptation results in up to ~ 30% more recognized syllables^14,15,21^. When the SNR is adaptively varied to estimate the SNR at 50% word recognition, speech reception thresholds (SRTs) can be up to 5 dB better when words are delayed 300 ms from the noise onset than when words and noise start at the same time^16,22,23^.

Hearing-impaired listeners vary widely in their ability to understand audible speech in noise (or speech in speech). Although previous studies have considered different predictors of this variability^11,24–29^, it has been neglected that HI listeners may adapt more or less to noise depending on the degree of hearing loss. Evidence exists that adaptation to noise in pure tone or AM detection is less for HI listeners than for NH listeners^30–35^. In AM detection, HI listeners show overall better AM detection thresholds than NH listeners, and do not benefit from adding an adapting noise (or precursor) before the AM tone, whereas NH listeners do^34^. The better AM thresholds are thought to be a consequence of HI listeners having more linear basilar membrane (BM) responses because of outer hair cell (OHC) loss, which results in an enhanced AM representation at the BM. The smaller adaptation for HI listeners has been interpreted to be a consequence of medial olivocochlear (MOC) efferent effects having a smaller range of action to linearize BM responses^34^.

Because speech recognition for HI listeners relies more on envelope (AM) cues than on temporal fine structure (TFS) cues^4,10,36^ and because HI listeners exhibit less than normal adaptation to noise in AM detection, adaptation to noise in speech recognition should be less for HI listeners than for NH listeners, either when speech is natural or processed (vocoded) to preserve envelope cues and discard TFS cues. To our knowledge, only Ben-David et al.^22^ have investigated adaptation to noise in speech recognition including a group of HI listeners. They showed that older HI listeners do not adapt to multitalker babble noise but adapt to steady noise as younger NH listeners do. However, it is uncertain whether these results are related to age, hearing loss, or the two factors. In addition, because Ben-David et al. aimed at investigating the effects of aging but not of hearing loss on adaptation to noise, listeners performed the experiment using their better ear, and the audiometric thresholds of their HI listeners did not differ much from those of their NH listeners [the thresholds of their HI listeners were ~ 10 dB HL at low-to-mid frequencies (mean across 0.5, 1, and 2 kHz) and ~ 32 dB HL at mid-to-high frequencies (mean across 3, 4, 6, and 8 kHz)]. Further research including participants with greater audiometric losses and controlling for the possible effect of age on adaptation is necessary to shed light on the impact of hearing loss on adaptation.

Here, we measured adaptation to noise for NH and HI listeners presented with natural and vocoded words in steady noise. We compared SRTs in noise for NH and HI listeners in different conditions (natural and vocoded words presented with and without an adapting precursor noise) to estimate the contribution of different factors (adaptation loss as well as the impaired access to speech envelope, TFS, or spectral cues) to the SRT loss of HI listeners. HI listeners were provided with frequency-specific, linear amplification to compensate for reduced audibility, and the possible effect of age on adaptation was controlled for statistically.

## Materials and methods

### Experimental design

A total of 25 HI listeners participated in the study. We measured SRTs (i.e., the SNR at 50% word recognition) for natural (unprocessed) and tone-vocoded words embedded in simultaneous speech-shaped noise (SSN). Words could be preceded or not by a 1-second-long noise precursor with identical spectrum and level as the simultaneous noise. Adaptation to noise was calculated as the improvement in SRT caused by the precursor relative to the no-precursor condition. The stimuli were presented monaurally and amplified using a software-based multichannel hearing-aid (HA), which provided frequency-specific linear amplification according to the National Acoustics Laboratory – Revised (NAL-R) gain prescription^37^. The results for HI listeners were compared with those of NH listeners published in a previous study^17^.

### Participants

Twenty-five HI listeners (9 men) participated in the experiment (mean age = 61.5 years; standard deviation (SD) = 9.3 years). The participants were native Spanish speakers, except for one of them, who was a native French speaker but had a high level of proficiency in Spanish.

**Fig. 1:**
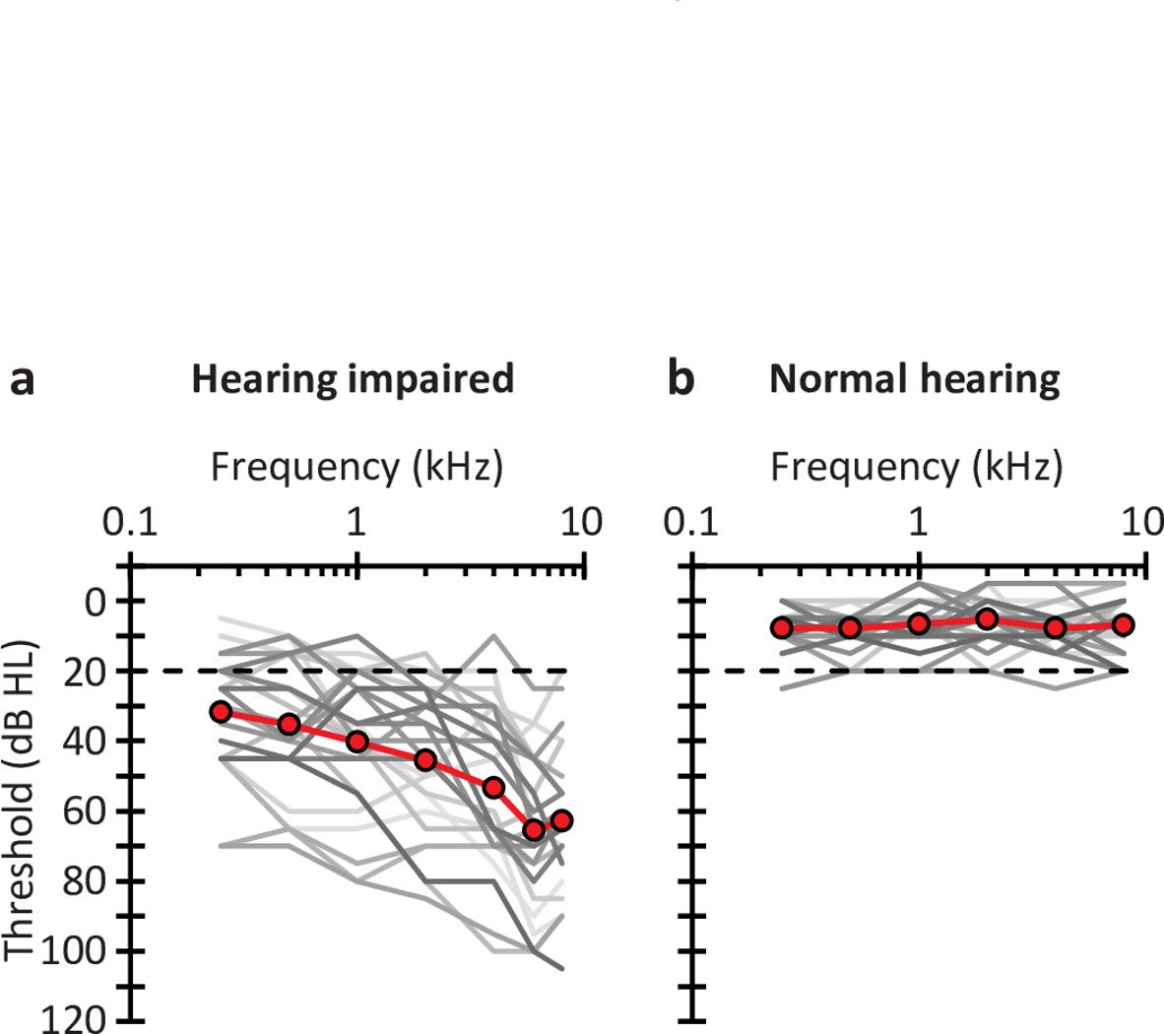
Audiometric thresholds for the study participants. (**A**) Air-conduction thresholds for the test ear of the 25 HI listeners. The line with symbols depicts the group mean audiogram; lines without symbols depict the audiograms of individual participants. The dashed line at 20 dB HL depicts the maximum level typically accepted for a person to be regarded as NH. (**B**) As panel (**A**) but for NH listeners (*N* = 28) (data from Marrufo-Pérez et al.^17^).

Air- and bone-conduction hearing thresholds were measured using a clinical audiometer (Interacoustics AD229e). Air-conduction thresholds were measured in the two ears at octave frequencies between 0.25 and 8.0 kHz, as well as at 6.0 kHz. All but two participants had symmetrical hearing loss (mean air-conduction threshold difference between the two ears < 15 dB at 0.5, 1, and 2 kHz, and < 30 dB at 4 and 6 kHz^38^). For participants with symmetrical hearing loss, the test ear was chosen at random (14 left, 9 right ears). The two participants with asymmetrical hearing loss were tested in the ear with the worst air-conduction thresholds (the right ear in the two cases). The audiogram for the test ear of the 25 HI participants is plotted in Fig. 1A. For further analyses, we computed the arithmetic mean of the air conduction thresholds at 0.5, 1, 2, and 4 kHz, hereinafter referred as the pure-tone average (PTA). The PTA thresholds ranged from 17.5 to 82.5 dB HL [mean = 43.7 dB HL; SD = 18.1 dB].

To confirm that participants had sensorineural hearing loss, we also measured their bone-conduction hearing thresholds at octave frequencies between 0.5 and 4.0 kHz for the participants with air-conduction PTA > 30 dB HL in the tested ear. The exception was one participant with an air-conduction PTA of 82.5 dB HL, for whom we did not measure bone-conduction thresholds. For participants with symmetrical hearing loss, bone-conduction thresholds were measured in one ear without masking the contralateral ear. The measured ear was chosen at random because we assumed that the interaural attenuation in bone conduction was zero or close to zero^39^. For the two participants with asymmetrical hearing loss, thresholds were measured in the test ear with masking in the contralateral ear. For all participants, their bone conduction PTAs ranged from 21.3 to 65 dB HL (mean = 40.8 dB HL; SD = 14.9 dB). This indicates that they had sensorineural hearing loss.

The results for HI listeners were compared with the results for NH listeners from a previous study^17^. The air-conduction audiograms for the test ear of the 28 NH listeners are shown in Fig. 1B. The PTA thresholds for this group ranged from 0 to 21.3 dB HL (mean = 6.9 dB HL; SD = 5 dB). NH listeners were younger (mean age = 26.6 years; SD = 8.3; min = 17; max = 56) than HI listeners were (mean age = 61.5 years; SD = 9.3; min = 39; max = 82). For that reason, the correlation between hearing loss and adaptation to noise was controlled for age as described later.

Participants were volunteers and not paid for their time. All of them signed an informed consent form before they could participate in the study. Methods were approved by the Ethics Committee of the University of Salamanca (Spain) and performed in accordance with the Declaration of Helsinki.

### Stimuli

#### Hearing-impaired listeners

SRTs for noise-masked words were measured monaurally in the presence and in the absence of a noise precursor. Both the simultaneous and precursor noises were steady, had a speech-shaped spectrum, and had a level of 65 dB SPL before HA processing. In the condition without precursor, the simultaneous masking noise started 50 ms before the word onset and finished 50 ms after the word offset. The precursor noise had a duration of 1 s. Both the precursor and simultaneous noise had 20-ms raised-cosine onset and offset ramps. The time gap between the precursor noise and the simultaneous masking noise was 0 ms. Different precursor and simultaneous noises were generated for each word presentation (i.e., noises were not ‘frozen’). Adaptation to noise was calculated as the improvement in SRT with the precursor relative to the no-precursor condition.

SRTs were measured for natural^40^ and tone-vocoded disyllabic words (one participant was tested only with natural words). The vocoder was applied to the words but not to the noise. The vocoder included a high-pass preemphasis filter (first-order Butterworth filter with a 3-dB cutoff frequency of 1.2 kHz); a bank of 12, sixth-order Butterworth band-pass filters whose 3-dB cutoff frequencies followed a modified logarithmic distribution between 100 and 8500 Hz (center frequencies from channel #1 to #12 equal to 149, 262, 408, 601, 855, 1191, 1638, 2234, 3029, 4091, 5511, and 7412 Hz); and envelope extraction via full-wave rectification and low-pass filtering (fourth-order Butterworth low-pass filter with a 3-dB cutoff frequency of 400 Hz). The envelope for each frequency channel was used to modulate a sinusoidal carrier at the channel center frequency, and the modulated signals were filtered again through the corresponding filter in the bank and samplewise added to obtain the vocoded speech.

Stimuli (speech and noise) were processed through a 12-channel, software-based HA implemented in the frequency domain in Matlab (The MathWorks, v2017a). The HA has been fully described elsewhere^13^ but was set with linear amplification according to the NAL-R gain prescription^37^.

#### Normal-hearing listeners

The results of the present study for HI listeners were compared with the results for NH listeners from a previous study^17^. The stimuli used in the two studies were similar except that a precursor noise 1.0 s in duration was used for HI listeners while for NH listeners words were delayed in the noise by 1.5 s. The difference in the preceding noise duration is not expected to affect the amount of adaptation because the time course of adaptation is ~ 350 ms and SRTs in noise do not improve further for noise-word onset delays beyond 600 ms. This time course is independent of age or hearing status^22^.

### Procedure

Phonetically balanced word lists^40^ were used to measure the SRTs. Before starting an SRT measurement, the experimenter chose one of the ten available lists at random. Each list contained 25 disyllabic words. Words from a given list were presented in random order across test conditions to minimize the possibility that participants remembered the words. To measure the SRT, the noise level was fixed. The speech level varied adaptively using a one-down, one-up adaptive rule, i.e., it decreased after a correct response and increased after an incorrect response. The SRT was thus defined as the SNR yielding 50% correct word recognition in the psychometric function^41^. The initial SNR was 10 dB. The speech level changed in 4-dB steps between words 1 and 8 and in 2-dB steps between words 9 and 25. The SRT was calculated as the mean of the SNRs for the final 15 words (the SNR for the 16th word was calculated and used in the SRT estimate but not actually presented). Feedback was not given to the participants on the correctness of their responses.

The SRTs with and without precursor were measured in pairs but in random order. An SRT was discarded immediately after the measurement, and a new SRT was measured when the SD within the measurement was greater than 3 dB. Three SRTs were obtained for each condition, but if the experimenter deemed that the across-measures SD was high (no objective criteria were used), a fourth pair of SRTs was obtained. The mean of the three or four SRTs was taken as the final SRT.

During the measurements, participants were seated in a double-wall sound attenuating booth and the presentation of each word was controlled by the experimenter, who was sitting outside the booth without visual interaction with the participant. Participants were asked to repeat each word into a microphone after hearing them or, if they did not recognize the word, they were asked to indicate so. The experimenter received the participant’s response through headphones and marked the word as correct or incorrect in the software. The word was scored as correct when the participant correctly repeated all its phonemes. A sound cue (1-kHz pure tone with 500 ms duration) was presented 500 ms before the stimulus onset to warn the listener about the stimulus presentation and to focus his/her attention on the speech recognition task. The level of the cue was 83 dB SPL for HI listeners and 66 dB SPL for NH listeners.

### Apparatus

The stimuli were presented through custom-made Matlab software (The MathWorks, v2017a) and played via an RME Fireface UCX soundcard at a sampling rate of 44.1 kHz and with 24-bit resolution. For NH listeners, stimuli were presented using insert earphones (ER2, Etymotic Research). The sound pressure levels (SPLs) were calibrated by inserting the earphones into a Zwislocki DB-100 coupler connected to a sound level meter (Brüel Kjaer, mod. 2238). For HI listeners, stimuli were presented using circumaural headphones (Sennheiser HD580), which provided higher output levels than did the insert earphones and prevented clipping from occurring when the hearing loss was severe. The SPLs were calibrated by placing the headphones on a KEMAR head (Knowles Electronics) equipped with a Zwislocki (Knowles Electronics DB-100) artificial ear connected to a sound level meter (Brüel & Kjaer 2238). For the two transducers, calibration was performed at 1 kHz and the obtained sensitivity was used at all other frequencies.

### Statistical analyses

All the statistical analyses were performed using IBM SPSS Statistics, version 28. The Shapiro-Wilk test of normality revealed that the data did not conform to a Gaussian distribution. Hence, nonparametric statistical tests were used to compare SRTs or adaptation to noise across participant groups (NH and HI listeners) or across conditions (no precursor/precursor) as well as to correlate different measures. We hypothesized that (1) SRTs would be better with than without the precursor noise, and (2) adaptation would be less for HI listeners than for NH listeners. Because these hypotheses were directional, we report one-tailed significance levels for all tests. An effect was regarded as statistically significant when the null hypotheses could be rejected with 95% confidence (*p* ≤ 0.05). No corrections for multiple comparisons were applied.

## Results

**Fig. 2:**
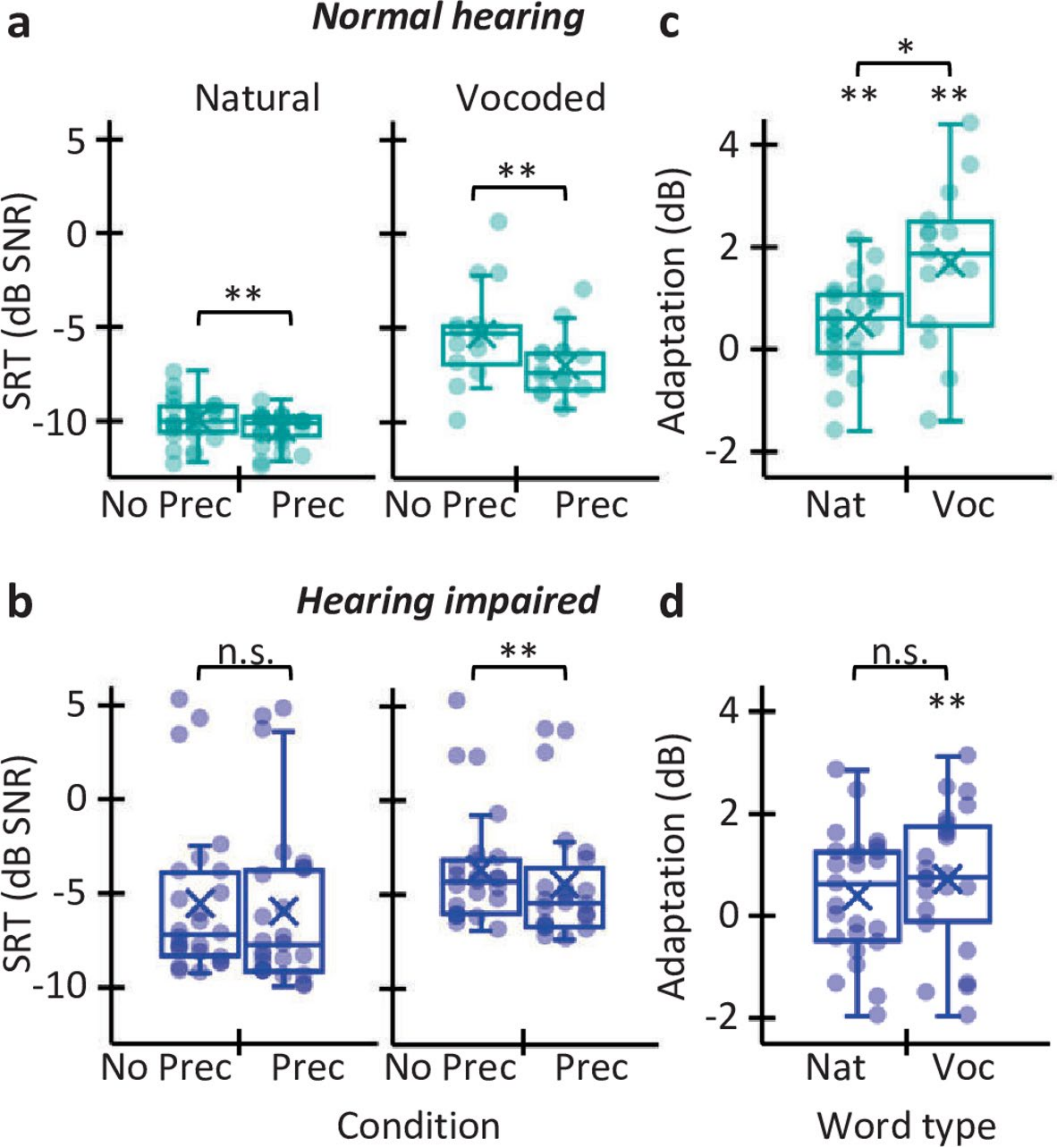
SRTs and adaptation to noise for NH and HI listeners. **(A)** SRTs for NH listeners presented with natural (*N* = 27) or vocoded (*N* = 15) words in steady noise. SRTs are shown without (‘No Prec’) and with (‘Prec’) steady noise precursor. Bottom, middle, and top lines in each box plot indicate the 25th, 50th (median), and 75th percentiles, respectively. Crosses represent mean scores. Dots illustrate individual scores. **(B)** As panel **(A)** but for HI listeners. The sample size was 25 and 24 participants for natural and vocoded words, respectively. **(C)** Adaptation to noise for NH listeners for natural and vocoded words. Positive values indicate better SRTs with than without a precursor. **(D)** As panel **(C)** but for HI listeners. *p < 0.05; **p ≤ 0.01.

**Fig. 3:**
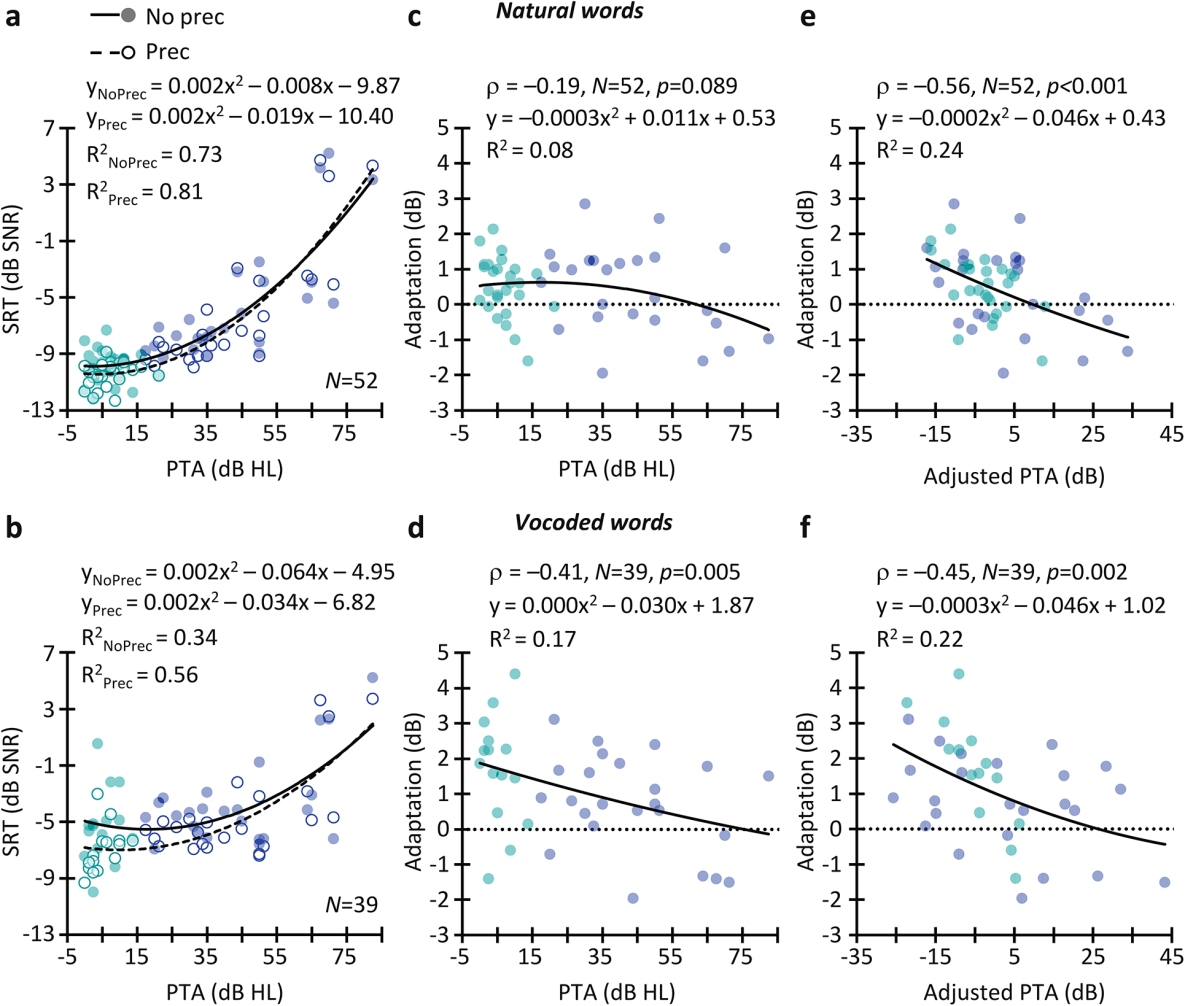
Relationship between hearing loss and adaptation to noise. **(A)** SRTs for natural words measured without (filled dots; solid line) and with a noise precursor (empty dots; dashed line) as a function of PTA (0.5–4 kHz). Green dots depict data for NH listeners, and blue dots depict data for HI listeners. Lines indicate quadratic regression fits to the data. **(B)** As A but for vocoded words. **(C)** Relationship between adaptation to noise for natural words and PTA. **(D)** As C but for vocoded words. **(E)** Relationship between adaptation to noise for natural words and PTA when the effects of age and SRT_NoPrec_ were removed from the PTA. **(F)** As E but for vocoded words.

Figure 2A shows the SRTs for NH listeners presented with natural and vocoded words, without and with precursor. Figure 2B shows the corresponding data for HI listeners. Let us first analyze the SRTs measured without the noise precursor to assess the effect of hearing loss on intelligibility independent of adaptation. In that case, SRTs were overall worse (higher) for HI listeners than for NH listeners, both for natural (Mann-Whitney U = 32.5, *p* < 0.001) and vocoded words (U = 119.0, *p* = 0.039). In addition, the SRTs worsened as hearing loss increased both for natural (Spearman Rho ρ = 0.74, *N* = 52, *p* < 0.001) and vocoded (ρ = 0.36, *N* = 39, *p* = 0.012) words (Fig. 3A). Because stimuli were presented to HI listeners through a HA with frequency-specific linear amplification^37^, the worse SRTs for HI listeners were unlikely related to audibility deficits. Instead, the results suggest that HI listeners were less able to use speech-related acoustic cues as hearing loss increases. The specific cues will be discussed later.

To investigate whether listeners showed adaptation to noise, Fig. 2A, B compares SRTs without and with the noise precursor. For NH listeners, the Wilcoxon signed rank test showed that the noise precursor improved SRTs for both natural (*N* = 27; Z= -2.73; *p* = 0.003) and vocoded (*N* = 15; Z= -3.0; *p* = 0.002) words, although adaptation was greater for vocoded words (*N* = 14; Z= -2.2; *p* = 0.013) (Fig. 2C). HI listeners did not show statistically significant adaptation for natural words (*N* = 25; Z= -1.5; *p* = 0.061) but they did show adaptation for vocoded words (*N* = 24; Z= -2.3; *p* = 0.010) (Fig. 2D). The amount of adaptation to noise was low overall and not significantly different for the two listener groups when listeners were presented with natural words (0.5 dB for NH; 0.4 dB for HI; Mann-Whitney U = 332.0; *p* = 0.460). In contrast, adaptation was greater for NH listeners (1.7 dB) than for HI listeners (0.7 dB) when they were presented with vocoded words (U = 113.0; *p* = 0.027).

Because HI listeners did not adapt to noise with natural words, and showed less than normal adaptation with vocoded words, the results show that hearing loss impairs adaptation to noise. To investigate this further, Fig. 3A, B shows the relationship between the PTA threshold and the SRTs measured with and without the noise precursor. Clearly, the effect of the precursor on SRT decreases as the PTA increases, indicating that hearing loss impairs adaptation to noise. [Similar results were found when the PTA threshold was calculated by averaging audiometric thresholds across all audiometric frequencies (0.25–8 kHz) (not shown)]. Figure 3C, D shows the amount of adaptation as a function of PTA. The correlation was not statistically significant for natural words (Spearman Rho ρ=-0.19, *N* = 52, *p* = 0.089) but it was significant for vocoded words (ρ=-0.41, *N* = 39, *p* = 0.005). For the two types of words, listeners no longer benefited from adaptation when the PTA is 70 dB HL or higher (i.e., the regression line approaches zero).

The relationship between PTA and adaptation might, however, be reflecting the effect of a mixture of variables because PTA is correlated with age (ρ = 0.705, *N* = 53, *p* < 0.001) as well as with the baseline SRT measured without the precursor (SRT_NoPrec_) (natural: ρ = 0.743, *N* = 52, *p <* 0.001; vocoded: ρ = 0.361, *N* = 39, *p =* 0.012). Age^22^ and the baseline SRT^16^ can affect the amount of adaptation. To investigate the specific impact of hearing loss on adaptation, we performed a semipartial correlation between PTA and adaptation to noise while controlling for the effects of age and SRT_NoPrec_ on the PTA. Spearman’s rank-order semipartial correlation revealed a correlation between PTA and adaptation to noise for natural (ρ*=*-0.56, *N* = 52, *p <* 0.001) and vocoded words (ρ=-0.45, *N* = 39, *p* = 0.002) (Fig. 3E, F). This finding suggests that hearing loss per se, rather than age or baseline intelligibility, degrades adaptation to noise.

Note that our sample includes 25 HI listeners but only four of them had severe or close to severe hearing loss (PTA ≥ 68 dB HL) (Fig. 3A-B). Three of those listeners showed significantly worse SRTs than the rest of the sample (Fig. 2B) and one might think that the relationships shown in Fig. 3A-B may disappear when the SRTs (~ 4 dB SNR) from those listeners are excluded. This, however, was not the case (supplementary Fig. S1). Moreover, the Spearman’s rank-order semipartial correlation between PTA and adaptation to noise remains significant for natural (ρ*=*-0.49, *N* = 49, *p <* 0.001) and vocoded words (ρ=-0.31, *N* = 36, *p* = 0.033) when the data for those three HI listeners are omitted (supplementary Fig. S1E, F). This corroborates that the results described above are not biased by those three participants.

## Discussion

The present study aimed to investigate the impact of hearing loss on the ability to adapt to noise during speech recognition. We found that, in aided conditions, the SRT for words in competition with SSN worsens with increasing hearing loss, both for natural and vocoded words. We also found that adaptation to noise decreases as hearing loss increases, both for natural and vocoded words. These relationships held when the effect of age on PTA was partialled out, which demonstrates that the loss of adaptation related to hearing loss and not to ageing.

### The loss of adaptation to noise adds to the loss of speech information

Previous studies have shown that hearing loss impairs the encoding of and thus the access to speech TFS^4,10,36,42,43^ and envelope information^42,44,45^. It also degrades the resolution of the long-term speech spectrum in the auditory system because of poor frequency selectivity^2,46–50^. In addition, hearing loss at frequencies higher than those commonly measured in the audiogram (8–20 kHz) contributes to impaired speech recognition in broadband noise^51^. Here, we demonstrate that hearing loss not only impairs access to speech acoustic cues, but also impairs the ability of listeners to adapt to noise.

**Fig. 4:**
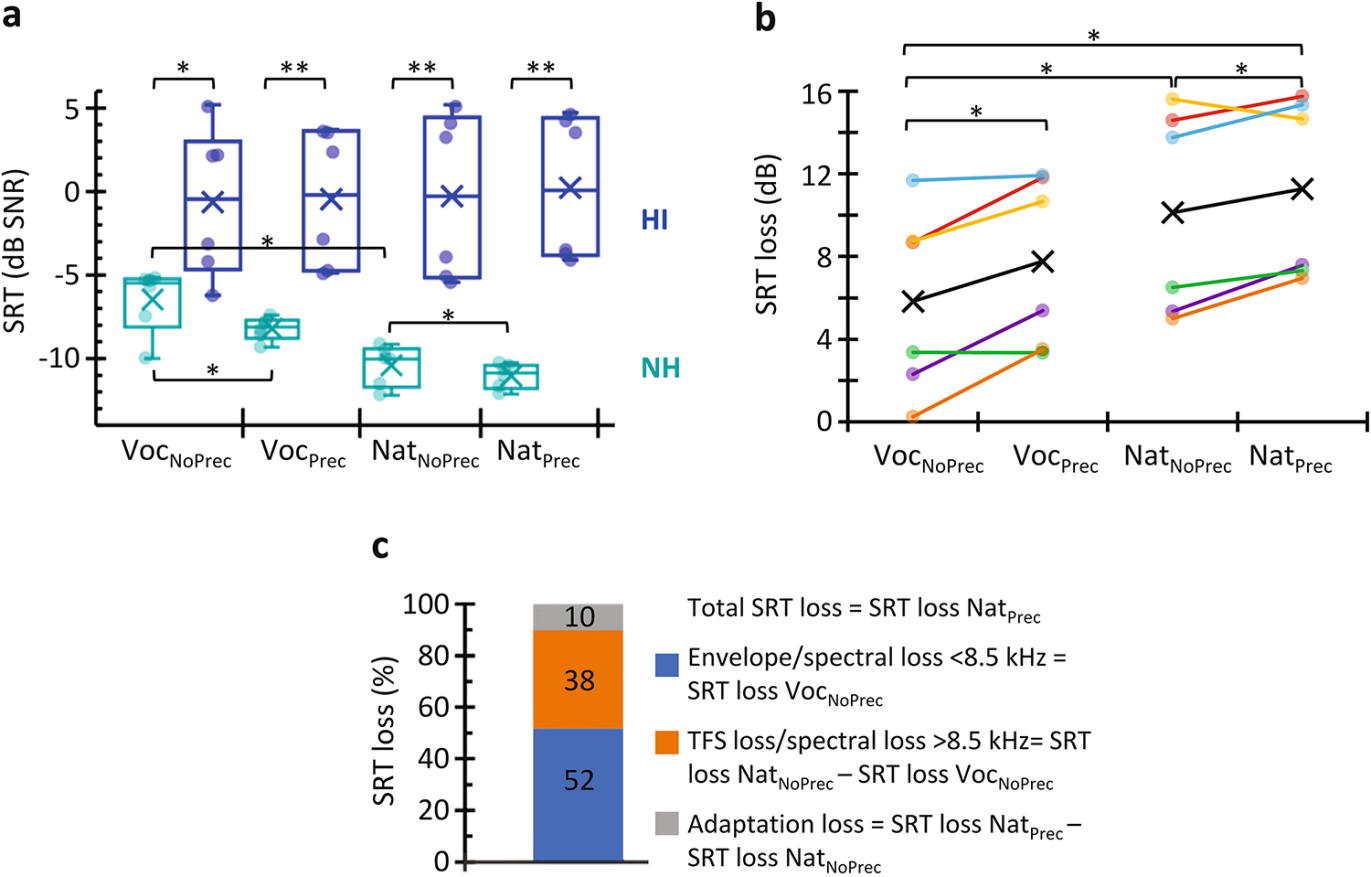
The contribution of different factors to the loss of intelligibility for HI listeners with 64 to 83 dB hearing loss. **(A)** SRTs for the six listeners with the best (NH, green) and the worst PTA thresholds (HI, blue) in the four experimental conditions of the study. *p < 0.05, **p ≤ 0.01 for Mann-Whitney test (across participants) or Wilcoxon test (across condition). **(B)** Difference between the SRTs for HI listeners and the mean SRT for NH listeners in the respective condition. Each color line depicts the results for one HI listener. Black line with crosses depicts the mean. **(C)** Contribution of the loss of speech cues and adaptation to noise to the total SRT loss.

Figure 4 illustrates the contribution of various factors to the loss of intelligibility for the present HI listeners with the greatest hearing loss (supplementary Fig. S2 shows similar data for participants with smaller losses). Panel A shows the SRTs for the six NH listeners with the best PTA thresholds (from 0 to 3 dB HL; mean = 1.7 dB HL) and for the six HI listeners with the worst PTA thresholds (from 64 to 83 dB HL; mean = 70.0 dB HL). Six listeners were chosen for each group as a compromise between showing the most extreme differences and not including only the participants with the worst and the best PTA. Figure 4A shows that NH listeners had better SRTs for natural than for vocoded speech and even better SRTs when they had the opportunity to adapt to the noise. In contrast, HI listeners exhibited worse SRTs than NH listeners (*p* < 0.05) and virtually constant SRTs across conditions (*p* ≥ 0.147). Figure 4B shows the SRT loss, which was calculated as the difference between the SRTs for individual HI listeners and the mean SRT for NH listeners in each condition. The largest SRT loss occurred for natural words with precursor (mean = 11.3 dB; Fig. 4B) presumably because in this condition HI listeners suffered from impaired access to all the possible speech cues (envelope, TFS, spectrum) plus a loss of adaptation. For convenience, we will refer to the largest SRT loss as ‘total’ SRT loss (Fig. 4C). To shed further light on the contribution of each factor to the total SRT loss we interpreted the results as follows. First, because the vocoder preserved only the speech envelope and spectral information below 8.5 kHz (the highest cutoff frequency of the filter bank in the vocoder), the worse SRTs for vocoded words for HI listeners than for NH listeners (Voc_NoPrec_ in Fig. 4A, B) reveal an impaired ability of HI listeners to use envelope and/or spectral information below 8.5 kHz. This impairment explained 52% of the total SRT loss (Fig. 4C). Second, because the recognition of natural speech depends on all the same cues as the recognition of vocoded speech plus TFS^36^ and high-frequency (> 8.5 kHz) information^51^, the difference between natural and vocoded words reveals the ability to use TFS and high-frequency spectral information. Because NH but not HI listeners showed better SRTs for natural words than for vocoded words (Voc_NoPrec_ vs. Nat_NoPrec_ in Fig. 4A), our results suggest that NH but not HI listeners benefit from adding TFS or high-frequency spectral information to speech. The lack of TFS and high-frequency spectral speech information explained 38% of the total SRT loss (Fig. 4C). Lastly, the improvement in SRT when a precursor is present shows the benefit of adaptation to noise. NH but not HI listeners showed adaptation (Fig. 4A), and the loss of adaptation explained the remaining 10% of the total SRT loss (Fig. 4C). Taken together, the present results show that HI listeners have difficulties recognizing audible speech in noisy conditions not only because they are less able to use/encode speech acoustic cues but also because they are less able to adapt to noisy backgrounds.

### On the reduced adaptation to noise for HI listeners

We found that hearing loss impairs adaptation to noise (Figs. 3 and 4). Multiple mechanisms can underlie this result^35,52^. Among them may be a decrease in the MOC reflex (MOCR)-mediated adjustment of the cochlear gain in the damaged cochlea. MOC fibers are reflexively activated with a time course of 277 ± 62 ms^53^. MOC efferents terminate upon OHCs, and their activation inhibits the gain of the cochlear amplifier, linearizing BM responses and reducing compression^54^. Jennings et al.^34^ reasoned that HI listeners do not show adaptation in AM detection because they have more linear BM responses and hence less MOCR-mediated BM linearization. A smaller adaptation to noise in speech recognition might also occur if the smaller MOCR-mediated BM linearization produces less enhancement of the speech envelope at the output of the BM responses in HI listeners. Some studies, however, have shown that adaptation to noise in speech recognition or AM detection can occur without MOCR effects^16,20^.

Another proposed mechanism for adaptation to noise is a shift in the dynamic range of auditory neurons toward the most common level in the noise preceding the word^16,17,20,21,35^. When auditory neurons are presented with low-varying-level noise, neurons shift their dynamic ranges toward the most common noise level so long as the level in question is above the neuron’s threshold. This adaptation increases a neuron’s sensitivity to changes in sound level^55,56^. The improvement in sensitivity to level changes, however, is smaller when the variance in the stimulus level is large^55,57^. Because audiometric hearing loss is often associated with the loss or dysfunction of inner hair cells (IHCs) and OHCs^58,59^, it is possible that the reduced auditory peripheral compression due to OHC loss causes the IHC receptor potential representation to fluctuate more than normal. This could result in neurons not having a prevailing level to adapt to, thus in less adaptation to noise for HI listeners than for NH listeners^17^. However, whether the inherent fluctuations of the steady noise at the output of a linear BM response are fluctuating enough to impair dynamic range adaptation to sound level statistics has yet to be determined.

A third potential mechanism is related to the disruption of the cues used by listeners to segregate streams, i.e., to segregate the speech and noise stimuli. Jennings et al.^60^ measured pure tone detection thresholds for short tones presented 2 or 197 ms after the onset of a 400 ms narrowband noise masker with a flattened or inherent fluctuating temporal envelope. They found that when the probe was delayed in the noise, detection thresholds improved for the tones presented in the flattened noise but worsened for the tones presented in the fluctuating noise. Jennings et al. reasoned that listeners rely on a temporal envelope-based cue to detect the probe and that the fluctuations in the preceding noise disrupt this cue. It has been shown that the amplitude fluctuations of steady noise can also impair speech recognition when presented simultaneously with the speech, presumably because it is difficult to distinguish the noise fluctuations from the amplitude fluctuations that convey speech information^61,62^. However, it is uncertain whether the precursor fluctuations can hinder speech recognition. If they did, the more linear BM responses of HI listeners would enhance the precursor noise fluctuations, making it harder for HI listeners to distinguish between noise and speech fluctuations, and resulting in less adaptation for HI listeners than for NH listeners. This, however, does not seem to be the case here because although SRTs were sometimes worse with the precursor, the worsening occurred for NH and HI listeners, so it did not seem to be related to the hearing loss (points below zero in Fig. 3C, D).

### Implications

The present study shows that adaptation to noise in speech recognition decreases with increasing hearing loss, even when the potential confounding effect of age is factored out. This finding is relevant in at least two ways. First, as explained earlier, HI listeners find it harder to recognize audible speech in noisy settings than NH listeners^1–5,8^. Impaired access to speech acoustic cues (envelope, TFS, spectrum) has been shown to contribute to impaired intelligibility. The present study reveals that the impact of hearing loss on speech recognition can be underestimated if adaptation is disregarded (Fig. 4). Second, research on the factors that can affect speech-in-noise intelligibility (for both NH and HI listeners) is often conducted disregarding adaptation or, more generally, temporal effects. Indeed, the speech-to-noise onset delay varies widely across studies (e.g., 500 ms in Johannesen et al.^24^; 3 s in Souza et al.^26^), sometimes it is not even reported^25,27–29^, and most times it is not justified. The present study suggests that the relevance of some factors may differ depending on the time when the speech is presented relative to the noise onset. For instance, cochlear mechanical dysfunction does not predict intelligibility in speech maskers for HI listeners^11^, but it could predict the SRT impairment related to a loss of adaptation to noise.

## Conclusions

We measured aided SRTs for words in competition with a steady SSN for listeners with different degrees of sensorineural hearing loss. Words were preceded or not by 1-second-long precursor adapting noise. We found that SRTs worsen and that adaptation to noise in speech recognition decreases with increasing hearing loss when speech is both natural and vocoded. Overall, the present results suggest that HI listeners exhibit impaired intelligibility in noisy environments not only because they are less able to use speech acoustic cues but also because they are less able to adapt to background noise. Impaired adaptation can contribute up to 10% of the SRT loss in HI listeners.

## Electronic supplementary material

Below is the link to the electronic supplementary material.

Supplementary Material 1

## Data Availability

All data are provided in the manuscript figures and supplementary information files. The actual data values will be provided by the corresponding author (E.A.L.P.) upon reasonable request.
