# Supplementary material for: Impaired noise adaptation contributes to speech intelligibility problems in people with hearing loss
Source: Sci Rep. 2024 Nov 20;14:28807. doi: 10.1038/s41598-024-80131-9 (PMC11579485; doi:10.1038/s41598-024-80131-9)
Supplement: Supplementary file 1 — Supplementary Material 1 [file 41598_2024_80131_MOESM1_ESM.pdf]

Impaired noise adaptation contributes to speech intelligibility problems in people with hearing loss

Miriam I. Marrufo-Pérez[1,2], Milagros J. Fumero[1,2], Almudena Eustaquio-Martín[1,2], Enrique A. Lopez-Poveda[1,2,3,*]

[1] *Instituto de Neurociencias de Castilla y León, Universidad de Salamanca, Salamanca, Spain.*

[2] *Instituto de Investigación Biomédica, Universidad de Salamanca, Salamanca, Spain.*

[3] *Departamento de Cirugía, Facultad de Medicina, Universidad de Salamanca, Salamanca, Spain.*

Supplementary Figure 1

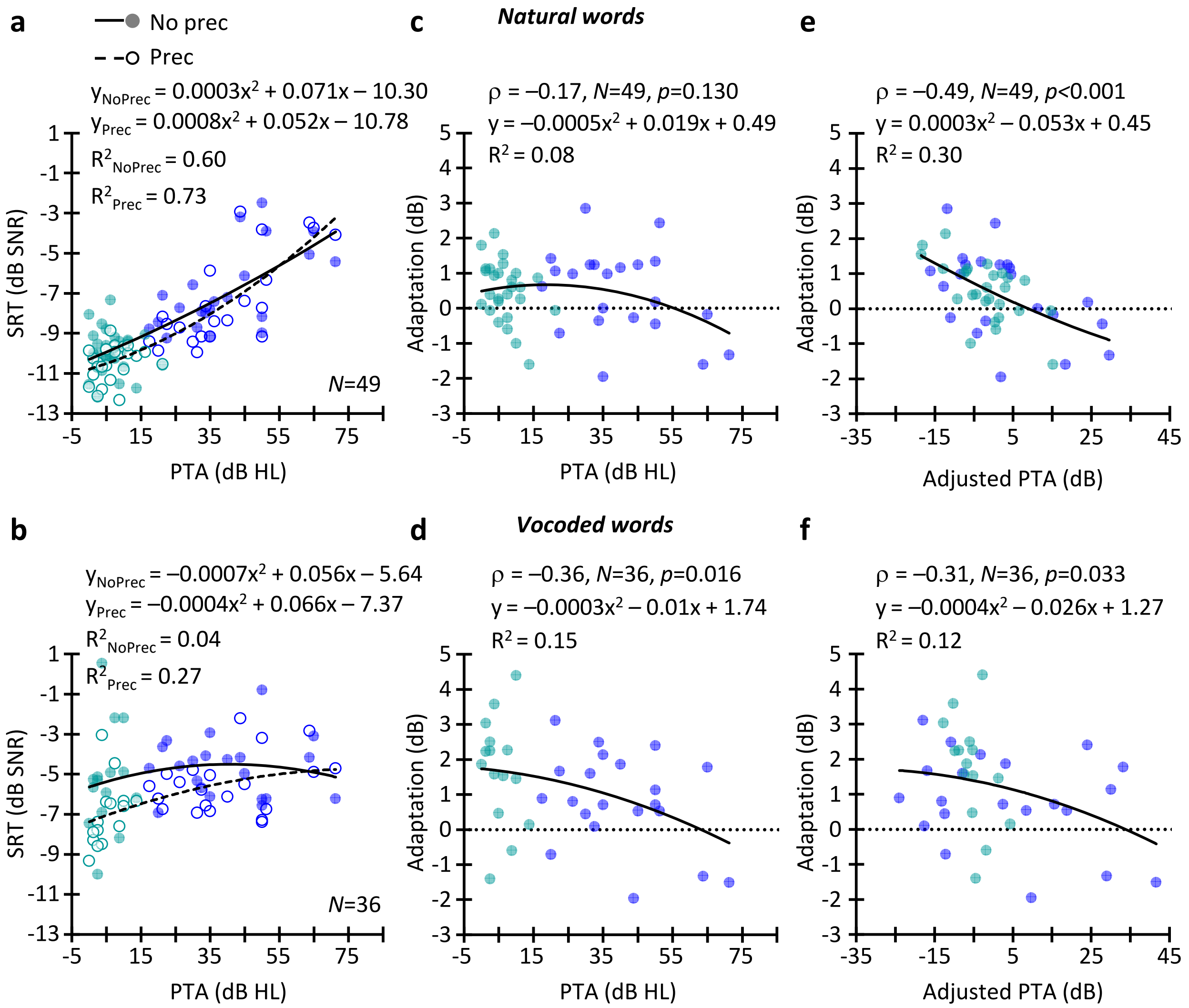

Supplementary Figure 1. Relationship between hearing loss and adaptation to noise when the three HI listeners with the highest SRTs were excluded from the analyses. The layout is the same as in Figure 3 from the manuscript.

Supplementary Figure 2

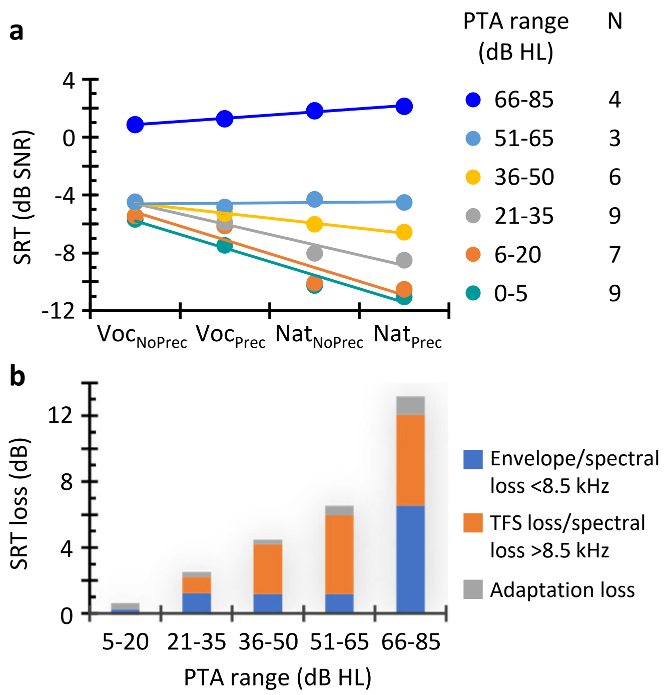

Supplementary Figure 2. The contribution of different factors to the loss of intelligibility for listeners with different degrees of hearing loss. A. SRTs for six participant groups with different PTA ranges (see legend). The mean PTA for each group is: 73 (blue), 60 (light blue), 46 (yellow), 30 (grey), 12 (orange), and 3 (green) dB HL. The number of participants included in each group is indicated at the right of the panel. Groups were designed in PTA steps of 15 dB (except for the 0-5 dB HL group) as a compromise to show the effect of different degrees of hearing loss on SRTs without having too many groups. To better illustrate the change in SRT across conditions, linear fits (lines) were made by arbitrarily numbering the conditions from 1 to 4. **B.** SRT loss for each group relative to the mean SRT for listeners with the best PTA (0-5 dB HL).
